# Single Cell Genomics meeting in Stockholm: from single cells to cell types

**DOI:** 10.1186/s13059-014-0496-x

**Published:** 2014-10-18

**Authors:** Antonio Scialdone, Kaia Achim, John C Marioni

**Affiliations:** Wellcome Trust Sanger Institute, Wellcome Trust Genome Campus, Hinxton, Cambridge, CB10 1SA UK; European Molecular Biology Laboratory - European Bioinformatics Institute (EMBL-EBI), Wellcome Trust Genome Campus, Hinxton, Cambridge, CB10 1SD UK; Developmental Biology Unit, European Molecular Biology Laboratory (EMBL), Meyerhofstraße 1, 69117 Heidelberg, Germany

## Abstract

A report on the second Single Cell Genomics conference held in Stockholm, Sweden, September 9–11, 2014.

The second Single Cell Genomics conference was held in Stockholm and hosted by the Karolinska Institute. The ‘Venice of the North’ was the setting for an exciting and intense meeting, with scientists from very different disciplines tackling the numerous challenges that single cell genomics presents.

During three days, 35 talks and more than 60 posters addressed many aspects of single cell genomics, from new experimental techniques to computational strategies for data analysis. We report on some of the main themes that emerged and, in our opinion, best illustrate the progress made and the new directions being undertaken by the field.

## Single cells, multiple -omics studies

Largely thanks to the use of microfluidics platforms for cell capture and reaction processing, single-cell RNA-seq has now become an established laboratory technique. Proving this, many high-quality single-cell RNA-seq data sets were presented, detecting typically around 7,000 species of transcripts per cell (2,000 to 10,000, depending on cell type and method), with the detection efficiency ranging from 10% up to 40%. Technical noise was typically controlled using synthetic spike-in RNA molecules and by using unique molecular identifiers to control for amplification biases.

Besides RNA-seq, single-cell genome sequencing (based on MDA (Multiple Displacement Amplification) or MALBAC (Multiple Annealing and Looping-based Amplification Cycles) techniques), methylation pattern analysis by bisulfite sequencing or by restriction enzyme mapping (Fuchou Tang, Peking University, China) and chromatin conformation mapping with single-cell Hi-C (Peter Fraser, The Babraham Institute, UK) are beginning to be explored.

However, the fraction of the genome assayed by single-cell genome sequencing still remains a problem. Nicholas Navin (MD Anderson Cancer Center, USA) showed how 91% coverage can be achieved with the nuc-seq method, which takes advantage of the duplicated genome content by using cells in G_2_/M phase. Another potential solution to the low-coverage issue was presented by Mike Stratton (Wellcome Trust Sanger Institute, UK), who used clonally derived organoids as a proxy of single cell genome sequencing. By tracking the distribution of 35 mutations across 25 organoids, Stratton and colleagues reconstructed the early cellular lineage that gave rise to the organoid seed cells. Interestingly, this work also indicated that different tissues display distinct ‘mutational signatures’, while distinct signatures are associated with the cell divisions in culture. Furthermore, it was exciting to hear about clinical applications of single cell genomics, as described in a keynote presentation by Sunney Xie (Harvard University, USA), who identified healthy (that is, mutation-free) oocytes for subsequent *in vitro* fertilization procedures by inferring the female pronucleus’ genome sequence from the sequence data derived from polar bodies.

The first successful combined analysis of DNA and RNA was also presented. A split-cell approach, in which the cell lysate was divided between different reactions, was described by Siddharth Dey (Hubrecht Institute, The Netherlands). Such ‘half-cell sequencing’ seemed to produce equally good data compared to more conventional single cell methods. From the perspective of quantifying protein expression, oligonucleotide-tagged antibodies have been exploited as a proxy for large-scale protein expression data (George Church, Harvard Medical School, USA).

## Capturing single cells in droplets

Clearly, the throughput of single cell analyses has dramatically increased over recent years, with up to thousands of single cells now being analyzed in a single experiment. This increase in throughput seems destined to increase further thanks to the emergence of new, advanced droplet-based microfluidics platforms. Now, in addition to the encapsulation of single cells into picoliter-volume droplets, each droplet can be barcoded and supplemented with unique molecular identifiers for transcript tagging, with magnetic beads facilitating further sample processing. Tens of thousands of droplets, each working as an individual chemical pico-reactor, can be generated and processed simultaneously. The droplet-based analysis of single cells was discussed by David Weitz (Harvard University, USA) and Evan Macosko (Broad Institute, USA). They reported good quality RNA-seq data, a reduced level of technical noise (probably resulting from reduced reaction volumes) and a dramatic reduction of costs per single-cell RNA-seq library. Other interesting applications of this technology, suggested by David Weitz, included single cell studies of virus or disease propagation by infection and re-infection of single cells (‘evolution-on-chip’), or single-cell screening for drugs or antibodies against infections.

## Exploring cell population structure

Single cell RNA-seq and single cell proteomics are very well suited to the identification of different cell types through the use of gene expression as a molecular signature. Indeed, the identification of novel cell types was one of the recurring themes of the conference.

Alexander van Oudenaarden (Hubrecht Institute, The Netherlands) described the different cell types identified in the intestinal crypt isolated from organoid cultures. This led to the identification of specific markers that proved useful for the targeted enrichment of rare cell types during subsequent sampling. Sten Linnarsson (Karolinska Institute, Sweden) gave evidence of new cell types in the mouse cerebral cortex. Other researchers presented the results of their cell-type hunting in the mouse cerebral cortex (Bosiljka Tasic, Allen Brain Institute, USA), the lung epithelium (Barbara Treutlein, Max Planck Institute, Germany), the hematopoietic system (Ido Amit, Weizmann Institute, Israel) and the olfactory epithelium (Peter Mombaerts, Max Planck Institute of Biophysics, Germany and Luís Saraiva, Wellcome Trust Sanger Institute, UK). The role of DNA methylation in preserving and transmitting information from mother to daughter cells and in maintaining cell-type-specific gene expression was the topic of Amos Tanay’s talk (Weizmann Institute, Israel). He described a comparison of the methylation profiles of clonally expanded stem cells with blood cells at various stages during differentiation.

Much discussion was devoted to the optimal methods for detecting different cell types. While standard techniques like hierarchical clustering and k-means are still widely used, effort is being invested to devise new methods that can better separate different cell types and identify rare cell populations; some of these methods were discussed by Alexander van Oudenaarden, Dana Pe’er (Columbia University, USA) and Sten Linnarsson. Increasing attention is also being drawn to the confounding effects that can hinder cell-type identification, such as technical noise and the cell cycle. Computational methods to identify and remove confounding effects were introduced in talks given by John Marioni (EMBL-EBI, UK) and Peter Kharchenko (Harvard Medical School, USA).

One important future challenge is to uncover the biological relevance of the putatively novel cell types identified to date. Ultimately, to answer this question, it may be necessary to integrate the RNA-seq data with information on the methylation states of DNA and possibly with functional studies.

## Spatial transcriptomics

Another clear trend was ‘spatial transcriptomics’. Several speakers described approaches for characterizing RNA expression *in situ* at the whole-transcriptome level: by multiplexed and/or sequential quantitative *in situ* hybridization (ISH) (Long Cai, Caltech, USA) or by positional tagging and RNA amplification directly on tissue section followed by standard Illumina sequencing (talks by Mats Nilsson, Stockholm University, Sweden and Joakim Lundeberg, KTH, Sweden). Distinct from direct on-tissue sequencing, the ‘RNA-tomography’ approach presented by Jan Junker (Hubrecht Institute, The Netherlands) uses systematic sequencing of thin sections from three stage-matched animals, each set of sections covering one of the three Cartesian axes, and subsequent three-dimensional reconstruction of acquired data points. While most of these methods offer high, even pseudo-cellular spatial resolution, the identification of transcript sets corresponding to a single cell still remains a challenge.

## From single cell analysis to mechanisms

Some engaging presentations showed the insights that single cell analysis can provide in understanding specific biological processes. Sunney Xie described work from his lab that shed light on the origin of transcriptional bursting in bacteria by using single molecule assay and single cell mRNA counting. A mix of single cell dynamics assays and synthetic biology was the strategy used to investigate the design of biological circuits by another keynote speaker, Michael Elowitz (Caltech, USA). In particular, Elowitz described how his lab studied the activation of regulatory proteins that follow pulsing dynamics, and the Notch signaling pathway, which was synthetically re-built using a bottom-up approach in order to gain insight into its function. Going back to single cell RNA-seq, Rickard Sandberg (Karolinska Institute, Sweden) described how monoallelic gene expression can be quantitatively explored by analyzing the transcriptome of single cells isolated from F_1_ hybrid mice. While these studies provide answers to some biological questions, they also underscore the need to develop and test quantitative models in order to better interpret the data and uncover the mechanisms underlying these processes.

## Conclusions

Overall, both talks and posters underlined the rapidly evolving nature of the single cell genomics field, especially on the experimental side. The field of single cell biology is expanding very quickly; so new exciting applications will surely be presented at next year’s single cell conference, which will take place in Utrecht.

## Abbreviations

MALBAC: Multiple Annealing and Looping-based Amplification Cycles
MDA: Multiple Displacement Amplification

